# An Overview on Collagen and Gelatin-Based Cryogels: Fabrication, Classification, Properties and Biomedical Applications

**DOI:** 10.3390/polym13142299

**Published:** 2021-07-14

**Authors:** Yujing He, Chunhua Wang, Chenzhi Wang, Yuanhang Xiao, Wei Lin

**Affiliations:** 1 Department of Biomass and Leather Engineering, Key Laboratory of Leather Chemistry and Engineering of Ministry of Education, Sichuan University, Chengdu 610065, China; heyujinghyj@foxmail.com (Y.H.); wczonly@foxmail.com (C.W.); yuanhang-1027@foxmail.com (Y.X.); 2 National Engineering Research Center of Clean Technology in Leather Industry, Sichuan University, Chengdu 610065, China

**Keywords:** collagen, gelatin, cryogels, fabrication, cross-linking, biomedical application

## Abstract

Decades of research into cryogels have resulted in the development of many types of cryogels for various applications. Collagen and gelatin possess nontoxicity, intrinsic gel-forming ability and physicochemical properties, and excellent biocompatibility and biodegradability, making them very desirable candidates for the fabrication of cryogels. Collagen-based cryogels (CBCs) and gelatin-based cryogels (GBCs) have been successfully applied as three-dimensional substrates for cell culture and have shown promise for biomedical use. A key point in the development of CBCs and GBCs is the quantitative and precise characterization of their properties and their correlation with preparation process and parameters, enabling these cryogels to be tuned to match engineering requirements. Great efforts have been devoted to fabricating these types of cryogels and exploring their potential biomedical application. However, to the best of our knowledge, no comprehensive overviews focused on CBCs and GBCs have been reported currently. In this review, we attempt to provide insight into the recent advances on such kinds of cryogels, including their fabrication methods and structural properties, as well as potential biomedical applications.

## 1. Introduction

Cryogels are the macroporous heterophase polymeric gels formed via the cryogenic treatment (freezing–thawing) of solvent-precursors systems [1,2]. Generally, the precursor systems consist of polymerizable macromonomers or low-molecular weight monomers and corresponding initiators; polymers and cross-linkers; polyelectrolytes and corresponding chelators; or polymeric precursors capable of formation of physical junction nodes under freezing conditions (such as gelatin and poly (vinyl alcohol) (PVA)) [3]. Unlike conventional gels, cryogels are three-dimensional network with interconnected macropores or supermacropores, enabling the unhindered diffusion of macromolecular solutes as well as the seeding, migration, and proliferation of cells [4,5]. Owing to their unique structure and characteristics, cryogels have been widely utilized in biomedical applications, such as cell or protein separation [6,7,8], tissue engineering [9,10,11], cell culture [12], and drug delivery [13,14].

Considering the biomedical application of cryogels, their biocompatibility is a major requirement from design to preparation. Collagen is the major protein in extracellular matrix (ECM) and widely exists in mammalian tissues, such as skin, tendon, cartilage, bone, as well as cornea [15]. Gelatin is a heterogeneous mixture of polypeptides obtained by the partial hydrolysis of collagen involving the destruction of cross-linkages between the polypeptide chains along with cleavage of some polypeptide bonds [16,17]. The appealing advantages of collagen and gelatin, such as high biocompatibility, biodegradability, and weak antigenicity, have made them desirable candidates for synthesis of cryogels with biomedical application.

In comparison with cryogels obtained from other synthetic polymers, the superiority of collagen-based cryogels (CBCs) and gelatin-based cryogels (GBCs) can be mainly summarized by taking into account three important aspects. Firstly, both collagen and gelatin are natural biopolymers, soluble in water and non-toxic to humans. Secondly, the arginine-glycine-aspartic acid (RGD) sequence present in their molecular structure can promote cell adhesion, as the RGD sequence can be recognized by the integrins (mainly α_5_β_1_ and α_v_β_3_) expressed on cell surface [18,19]. Thirdly, the same RGD structural features represent the target sequences of matrix metalloproteinases (MMPs) which make both collagen and gelatin easily biodegradable [20]. For the above reasons, CBCs and GBCs have been considered as promising materials in biomedical fields, particularly in cell culture [21,22], tissue engineering [23,24,25,26], drug delivery [27,28,29], skin wound repair [30], etc.

Although numerous articles focused on cryogels have been published, to the best of our knowledge, no systematic overviews about CBCs and GBCs have been reported to date. Hence, for a more comprehensive understanding of CBCs and GBCs, an overview of their structural basis and functions are presented. Specifically, the fabrications, classifications, properties, and biomedical applications of CBCs and GBCs, as well as their future perspectives, are summarized and discussed in this review.

## 2. Fabrication, Classifications, and Properties of CBCs and GBCs

### 2.1. Fabrication

**Figure 1:**
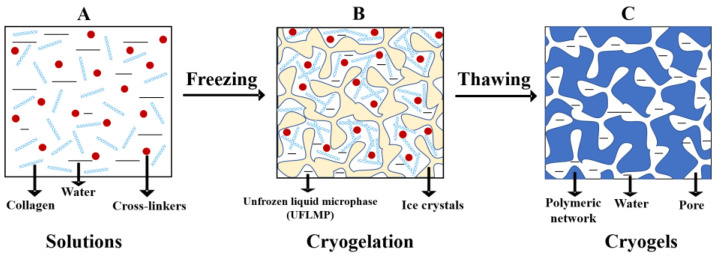
Collagen-based cryogels (CBCs) formation schematically illustrated. (**A**): initial collagen solution containing cross-linkers; (**B**): cryogelation process: the gelation occurs at the UFLMP at sub-zero temperatures and the formation of cryogel walls; (**C**): macroporous cryogel in native hydration state after thawing.

The formation of CBCs and GBCs is schematically presented in Figure 1 (taking the preparation of CBCs as an example). During the preparation process, ice crystals form and act as porogens by freezing the solutions containing collagen/gelatin and cross-linkers at a moderate negative temperature. Thus, the shape and size of the pores in cryogels are mainly controlled by the formed crystals. Meanwhile, collagen/gelatin, as well as cross-linkers are squeezed into an unfrozen phase between ice crystals and gelation reactions take place [31]. After thawing of the freezing system at room temperature, the CBCs and GBCs cryogels with interconnected macropores are produced.

It is worth noting that the unfrozen fraction in frozen systems is called unfrozen liquid microphase (UFLMP, shown in Figure 1B) [31,32]. Within the volume of UFLMP, the concentration of collagen or gelatin and cross-linkers increases significantly [2]. The phenomenon of local solutes-enrichment in UFLMP is known as cryoconcentration effects [33]. Interestingly, such cryoconcentration effects can not only completely counterbalance the negative impacts caused by low temperature, but also considerably reduce the critical concentration of gelation (CCG) of the precursors, becoming the main driving force for the cryogels formation [31,32,33]. In our previous study, we found that the sponge-like cryogels were produced when storing the solutions of collagen and dialdehyde starch (DAS) at −15 °C for 72 h, whereas no bulk gels were formed when storing it at 4 °C for 3 d, or even longer [34]. These experimental facts confirm the favorable role of cryoconcentration effects in the formation of cryogels.

**Figure 2:**
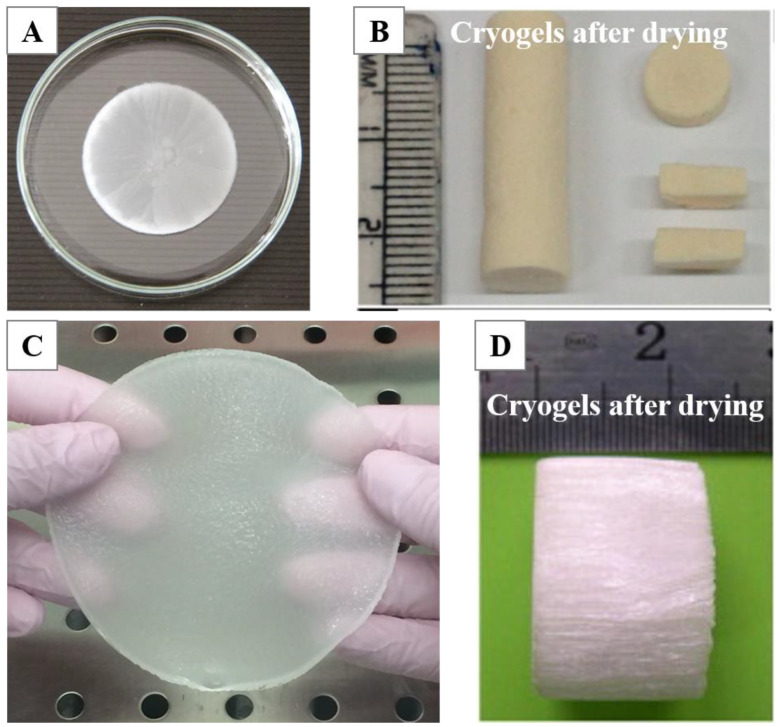
Images of CBCs and GBCs fabricated in different morphologies: membranes (**A**) [41], monoliths/discs (**B**) [9], sheets (**C**) [42], and cylinder (**D**) [38]. Reprinted from refs. [9,38,41,42] with permission, © ACS, Elsevier and De Gruyter.

The earliest studies of CBCs and GBCs commenced in the 2000 and 1980, respectively, and the different freezing methods were gradually developed [1,35]. The freezing–thawing methods of CBCs and GBCs primarily include one-step freezing–thawing, multiple freezing–thawing [36], and directional freezing [37,38]. The multiple freezing–thawing is the most widely used method, as it can markedly improve the mechanical properties of cryogels. Our current study shows that the uniformity, transparency, and mechanical strength of collagen cryogels cross-linked by aldehyde-poly (ethylene glycol) (PEG) are improved with the increasing freezing–thawing cycles (data unpublished). Schacht et al. also observed the same phenomenon for gelatin cryogels [36]. Although the reason for this is not very clear, it is likely that additional cross-linking is formed during repeated freezing–thawing processing. With an increase in the freezing incubation time, a more stable conformation is possibly formed, contributing to the enhanced mechanical property [36]. The directional freezing technique is commonly used to prepare CBCs and GBCs with anisotropic porous structures [37,38]. The pores along the freezing direction are parallel microtubules, while the pores perpendicular to the freezing direction appear as a honeycomb-like structure [39]. Moreover, the directional freezing can also lead to the anisotropic mechanical strength of cryogels [39,40]. According to different application requirements, CBCs and GBCs can be fabricated in different shapes, such as membrane (Figure 2A) [41], monoliths/discs (Figure 2B) [9], sheet (Figure 2C) [42], cylinder (Figure 2D) [38], etc. 

### 2.2. Classifications 

To toughen CBCs and GBCs, cross-linkers are highly desirable. According to the cross-linking mechanisms, CBCs and GBCs can be divided into two types, i.e., physically cross-linked and chemically (covalently) cross-linked, and will be briefly discussed in the following.

#### 2.2.1. Physical/Noncovalent Cross-Linking

Physically cross-linked CBCs and GBCs are formed only by physical interactions, such as hydrogen bonds, hydrophobic interactions, chains entanglement, etc. [35,36]. Such kinds of cryogels generally exhibit poor thermal stability, rapid degradation rate, and low mechanical strength, which limit their practical applications. 

#### 2.2.2. Covalent/Chemical Cross-Linking 

As for covalent crosslinking, the active groups on collagen and gelatin side chains, such as –NH_2_, –COOH, and –OH, provide desirable crosslinking sites. Normally, the covalently crosslinked CBCs and GBCs display markedly enhanced mechanical properties and thermal stability compared to the physically cross-linked ones. Moreover, based on the peculiarities of cross-linkers, CBCs and GBCs can be further classified into four types: crosslinked by small-molecule crosslinkers, by macromolecular crosslinkers and cryogels obtained via enzymatically mediated cross-linking or free-radical polymerization. 

##### Cross-Linking via Small-Molecule Cross-Linkers 

Currently, the common small-molecule cross-linkers used to prepare collagen and gelatin-based materials are aldehydes [43], 1-ethyl-3-(3-dimethyl aminopropyl) carbodiimide/N -hydroxysuccinimide (EDC/NHS) [44], and genipin [45].

**Figure 3:**
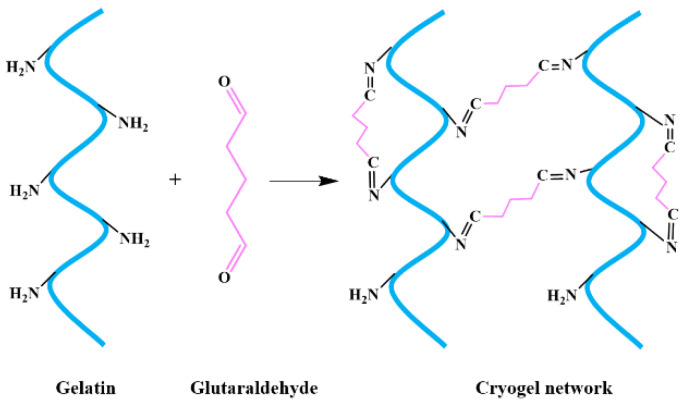
The reaction mechanism for the cross-linking of gelatin with glutaraldehyde.

As for aldehyde-type crosslinkers, glutaraldehyde is the most widely used cross-linking agent for collagen and gelatin, as its toxicity is lower than formaldehyde [46]. Additionally, previous studies have demonstrated that the Schiff’s base reaction between collagen/gelatin and glutaraldehyde could proceed in moderately frozen states [42,47]. The reaction mechanism of collagen and gelatin with glutaraldehyde is shown in Figure 3 (taking gelatin as an example) [48]. The mechanical strength of CBCs and GBCs are improved due to the formation of aldimine linkage (CH=N). Studies show that the compressive strength of GBCs exhibit an increasing trend with the increasing glutaraldehyde concentration [38,49,50]. It is worth noting that the residual glutaraldehyde in these cryogels has potential toxicity, which can influence the biocompatibility of cryogels, and thus limits their biomedical applications [51].

**Figure 4:**
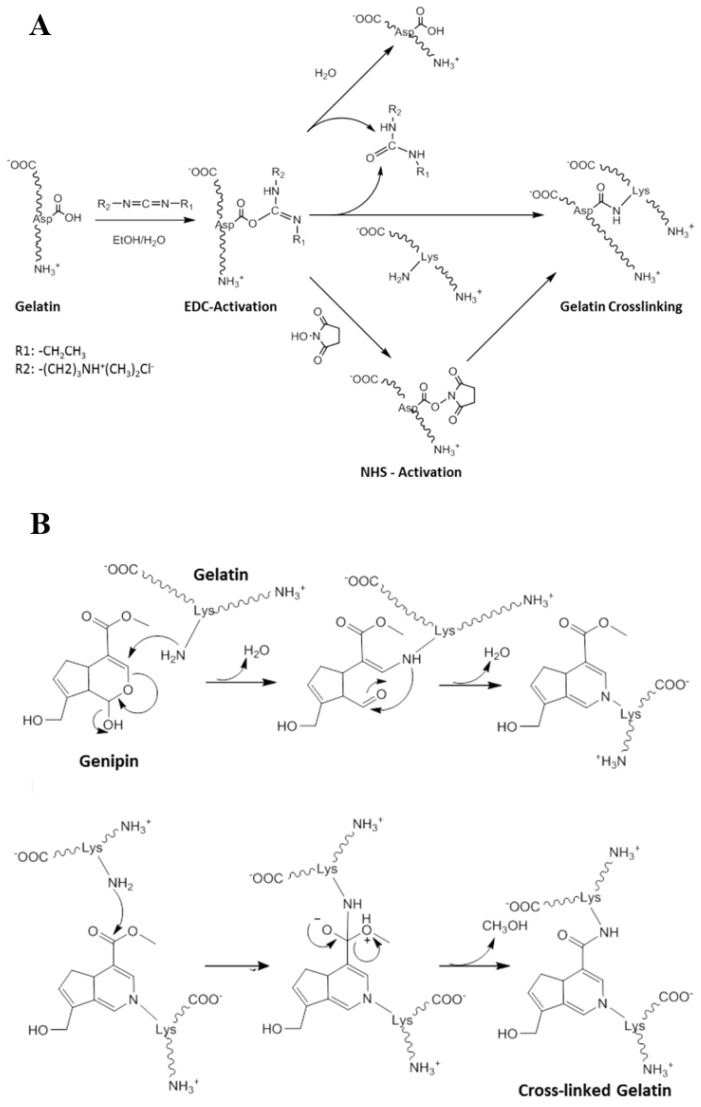
The reaction mechanism of gelatin with EDC/NHS (**A**) [53], and genipin (**B**) [53]. Reprinted from ref. [53] with permission, © MDPI.

Another commonly used small-molecule cross-linker for collagen and gelatin is 1-ethyl-3-(3-dimethyl aminopropyl) carbodiimide (EDC), which generally needs to be used in combination with N-hydroxysuccinimide (NHS) to improve the crosslinking efficiency [52]. The cross-linking mechanism of collagen and gelatin with EDC/NHS is schematically depicted in Figure 4A (taking gelatin as an example) [53]. Gorgieva et al. found that the thermal stability of gelatin cryogels were enhanced with the increasing EDC content [28]. For collagen/gelatin cryogels cross-linked by EDC/NHS, both the by-products and the remaining EDC and NHS can dissolve in water. Thus, they are easy to remove via repeated washing with water [52,53]. Therefore, the biocompatibility of CBCs and GBCs crosslinked by EDC/NHS is better than glutaraldehyde crosslinked gels [44].

In addition to glutaraldehyde and EDC/NHS, genipin, a natural product obtained from gardenia fruits, can also act as cross-linker to prepare CBCs and GBCs [54]. The reaction mechanism of genipin with collagen and gelatin is shown in Figure 4B (taking gelatin as an example) [45]. The main advantage of genipin is its natural origin and non-toxicity [55,56]. Thus, the cryogels cross-linked by genipin possess excellent biocompatibility. However, the price of genipin is relatively expensive compared to glutaraldehyde and EDC/NHS [55]. Moreover, the resulting cryogels exhibit dark blue appearance, which also limits its wide application [57].

##### Enzymes-Mediated Cross-Linking

Enzymes, as natural proteins, have become a novel type of crosslinker for preparing collagen and gelatin hydrogels, owing to their prominent reaction specificity [58,59]. Studies have shown that collagen and gelatin-based materials cross-linked by transglutaminase (TGase) possess more stable structure and stronger mechanical properties when compared with other enzymes (such as tyrosinases and horseradish peroxidases) cross-linked gels [60]. Due to its good biocompatibility, TGase is a widely used enzyme cross-linker for CBCs and GBCs [60,61,62].

For example, Kirsebom et al. used TGase as a cross-linker to mediate the reaction between the γ-carboxyamide groups present in glutamine and the ε-amino groups of lysine residues in gelatin to prepare gelatin cryogels [61]. They observed that no cryogels were produced after incubation the gelatin solution without TGase at −12 °C for 14 d. Although enzymes are non-toxic, the reaction rate mediated by enzymes is very slow in frozen state and requires a long time to form cryogels [61].

##### Cross-Linking via Macromolecular Cross-Linkers 

Although small-molecule aldehydes are the most effective cross-linkers for collagen and gelatin, the toxicity of free aldehydes can lead to undesirable side effects, such as inflammatory response, induction of calcification, and local cytotoxicity [63]. Given the abovementioned disadvantage of small-molecule aldehydes and the advantage of fast reaction rate of amino groups with aldehyde groups, the natural polysaccharides derivatives with dialdehyde groups such as dialdehyde starch (DAS) [34], oxidized dextran [64], dialdehyde carboxymethyl cellulose (DCMC) [63], have attracted increasing attention for the preparation of CBCs and GBCs.

**Figure 5:**
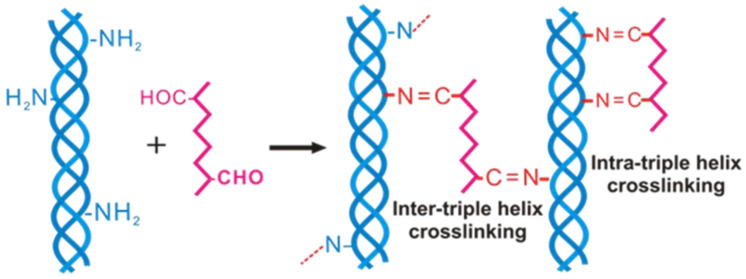
The cross-linking mechanism of collagen with DAS [34]. Reprinted from ref. [34] with permission, © Wiley.

In our previous study, we fabricated collagen cryogels using DAS as a cross-linker, and found that the denaturation temperature of cryogels could increase by 10–15 °C with the addition of DAS (the reaction mechanism of collagen with DAS shown in Figure 5) [34]. In 2015, we synthesized DCMC and utilized it as a macromolecular cross-linker for the preparation of collagen cryogels [63]. Results showed that the addition of a very small content of DMCM can apparently increase the denaturation temperature of collagen [63]. The thermal stability of collagen cryogels could increase by 4–20 °C when the content of DMCM increased from 0.02 wt% to 0.1 wt%. The reaction mechanism of collagen with DCMC is similar to that with DAS. As all the collagen cryogels cross-linked by DAS or DCMC exhibit a yellow appearance, we currently fabricated aldehyde terminated PEG (aldehyde-PEG), and used it as a novel cross-link agent to prepare collagen cryogels. As expected, the colorless cryogels were obtained and related research is underway.

Oxidized dextran is a macromolecular cross-linker used to prepare GBCs currently. Inci et al. and Berillo et al. investigated the physicochemical properties of gelatin cryogels cross-linked by oxidized dextran [64,65]. They found that the mechanical properties of gelatin cryogels can be further improved due to the crosslinking reaction between amino groups belonging to gelatin and the aldehyde groups of oxidized dextran. However, to the best of our knowledge, there are no publications about using DCMC, DAS, and aldehyde-PEG as macromolecular crosslinkers to prepare GBCs. Although the reaction mechanism of DCMC, DAS, and aldehyde-PEG with gelatin is similar to that with collagen, the influence of these cross-linkers on the properties of GBCs remains to be investigated.

Using macromolecular crosslinkers such as DCMC, DAS, and aldehyde-PEG can avoid the toxic effects produced by small aldehyde molecules, such as formaldehyde and glutaraldehyde. In fact, considering the biomedical applications, the abovementioned macromolecular crosslinkers could be a more desirable choice for cryogels fabrication compared to the small molecule ones.

##### Free-Radical Cross-Linking Polymerization

**Figure 6:**
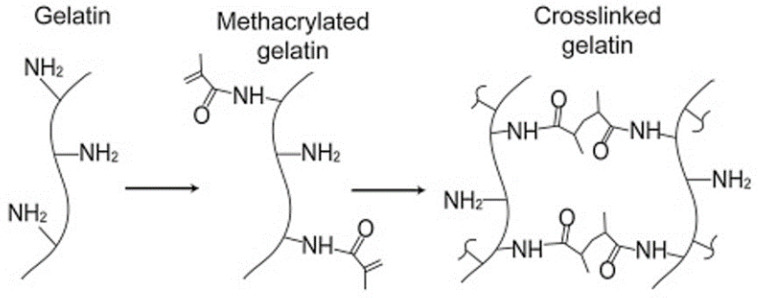
The mechanisms of free-radical polymerization for methacrylated gelatin (GelMA) [66]. Reprinted from ref. [66] with permission, © Elsevier.

CBCs and GBCs formed by using small molecule aldehydes or aldehyde-functionalized polysaccharides as cross-linkers display a low shelf-life and easily to turn yellow. The main reason for this is that the formed Schiff’s base is an unstable structure. The expensive price of genipin and enzymes limits their practical applications. Recently, the emerging strategy for the preparation of GBCs is free-radical polymerization. This cross-linking strategy is appropriate for the functional derivatives of gelatin such as methacrylated gelatin (GelMA) (the mechanism of free-radical polymerization for GelMA is shown in Figure 6).

GelMA was first synthesized in 2000 by Van den Bulcke through the reaction of methacrylic anhydride (MA) with the primary amines (lysine and hydroxyl lysine) of gelatin in phosphate buffered saline (pH = 7.4) at 50 °C [67]. The presence of vinyl group in the molecular structure of GelMA makes it polymerizable, while at the same time retaining its excellent biocompatibility and hydrophilicity [68,69]. GBCs can be obtained via free radical polymerization of GelMA triggered by redox initiator, UV irradiation, γ-irradiation, e-beam irradiation, etc. [67,70].

Specifically, Kwon’s group and Park’s group prepared gelatin cryogels via cryopolymerization of GelMA using ammonium persulfate (APS)/tetramethylethylenediamine (TEMED) as redox-initiator system at −20 °C [71,72]. By employing the same initiator system, Han et al. fabricated gelatin cryogels and investigated their mechanical properties [73]. They found that the compressive modulus of gelatin cryogels formed with 20% *w/v* GelMA can reach approximately 13 kPa [73]. Another study conducted by Koshy et al. showed that the gelatin cryogels with 1.0% GelMA possess good shape recovery ability, whereas the cryogels obtained with higher concentrations of GelMA are brittle [66]. In another study, Vlierberghe et al. fabricated gelatin cryogels through UV initiated free-radical polymerization of GelMA in the presence of photoinitiator Irgacure 2959 [74].

The free-radical polymerization method avoids the addition of chemical cross-linkers, thus eliminating their side effects. Previous study has demonstrated that gelatin-based hydrogels formed by free-radical polymerization display better biocompatibility than those formed by using glutaraldehyde as cross-linker [75].

#### 2.2.3. Cryogels Based on Collagen/Gelatin

In order to obtain the cryogels with desired mechanical properties or other specific functions, collagen and gelatin are usually covalently cross-linked with other synthetic polymers, natural polymers, and inorganic nanoparticles. Table 1 summarizes typical cryogels based on collagen and gelatin reported by the literature.

The effects of synthetic polymers, e.g., poly (vinyl alcohol) (PVA) [76,77], poly(acrylonitrile) (PAN) [78], polypyrrole (PPY) [79], poly(*N*-isopropylacrylamide) (PNIPAAm) [80], and poly (ε-caprolactone) (PCL) [106], on the properties of collagen or gelatin cryogels have been studied. For example, Jain et al. found that the incorporation of PAN could improve the mechanical properties of gelatin cryogels [78]. The Young’s modulus of gelatin/PAN cryogels increased by 696 kPa when the ratios of acrylonitrile to gelatin increased from 2:1 (123 kPa) to 5:1 (819 kPa). Tripathi et al. found that agarose/gelatin cryogels exhibited good elastic and mechanical strength [81]. Thus, no cracking took place when applying deformational stress on these systems. Milakin et al. observed that gelatin cryogel containing PPY not only had higher thermal stability than pristine gelatin cryogel, but also had electrical conductivity and antibacterial properties [79]. By using thermo-responsive characteristics of PNIPAAm, Sarkar et al. combined gelatin with PNIPAAm to prepare gelatin/PNIPAAm cryogels which also have thermo-responsive behavior [80]. However, the low biocompatibility and non-biodegradability of those synthetic polymers limit wide application of the corresponding CBCs and GBCs.

Natural polymers have been utilized to synthesize cryogels based on collagen or gelatin in recent years due to their excellent biocompatibility and biodegradability. For example, Kathuria et al. reported the formation of gelatin/chitosan cryogels [50]. An increased mechanical strength was observed for gelatin/chitosan cryogels compared to pure gelatin cryogels. Huang and co-workers have obtained similar results [82]. Heparin, as a sulfated glycosaminoglycan, and one of the components of the extracellular matrix (ECM), has been recently incorporated into gelatin to prepare cryogels [90]. The addition of heparin increased the extent of cross-linking, thus resulting in a high Young’s modulus of gelatin/heparin cryogels. Moreover, nanocellulose, fibrinogen, and carrageenan have also been reported to improve the mechanical properties of collagen or gelatin cryogels [27,49,86].

### 2.3. Properties of CBCs and GBCs

Cryogels properties, such as pore structure, mechanical strength, swelling ratio, biocompatibility, biodegradability, etc., play an important role from practical applications standpoint. For instance, when applied in the fields of bioseparation and wastewater treatment, highly interconnected macroporous structure is required to support the diffusion of oxygen and nutrients, and to guarantee the drainage of waste products from the matrix. In cell culture and tissue engineering, the porosity and pore interconnectivity is crucial, as it may alter cell adhesion, growth, and proliferation [74]. Gibson’s group and Huang’s group have been demonstrated the influence of pore size on cell adhesion and growth [107,108]. Therefore, the properties of pores, swelling behavior, and biocompatibility of collagen/gelatin cryogels are discussed in the following section.

#### 2.3.1. Pore Properties 

Compared with traditional hydrogels, CBCs and GBCs possess a relatively larger pore diameter and higher pore interconnectivity. The porosity, pore size, and pore morphology of CBCs and GBCs can be adjusted by varying the preparation parameters, such as component concentration, freezing temperature, and freezing rate. Vlierberghe et al. investigated the effect of gelatin content and freezing rate on the pore size of gelatin cryogels [74]. Their results showed that both the porosity and the pore size decreased with increasing the amount of gelatin. The reason for this is that a higher gelatin concentration in solutions could result in an increasing nucleation rate, and thus a larger number of pores was consequently obtained [74]. Another reason is that the more concentrated gelatin could possess a decreased heat and polypeptide transfer, with a direct result in smaller pores [74]. Vlierberghe et al. also found that the pore size of cryogels decreased with an increasing freezing rate [74]. However, it seems that the freezing rate does not affect the pore interconnection of collagen or gelatin cryogels. This is as the slower freezing rate leads to the lower nucleation rate, and thus results in a lower amount of lager pores [74]. The freezing temperature gradient mainly affects the pore geometry of CBCs and GBCs, leading to a transversal pore channels in the direction of heat transfer.

#### 2.3.2. Swelling Properties

Different from the morphology of collagen and gelatin-based hydrogels, the typical characteristic of cryogels is their sponge-like structures and interconnected systems of macropores. This difference may be mainly caused by cryogenic treatment. In other words, in the process of cryogels preparation, water forms irregular ice crystals, and meanwhile serves as the template for the formation of polymer network. In addition, the cross-linking reaction of collagen/gelatin with cross-linker in the UFLMP occurs, resulting in a highly cross-linked and 3D dense polymer network structure.

Analogous to other sponge-like cryogenically structured gel matrices, most water in these 3D systems can be separated out by applying a certain stress [3]. Our previous study, published in 2010, showed that the water in collagen cryogels are made up of bound water and free water [34]. The former is generally immobilized in the polymer network, and its proportion with respect to the total amounts of water is much less than free water, which fills the macropores of cryogels and can be squeezed out from cryogels. Savina’s results also showed that the water in gelatin cryogels is mainly free water [109]. The primary reason is the large and highly interconnected pores of the cryogels.

Due to interconnected pores of the cryogels, CBCs and GBCs have good permeability. They can quickly absorb liquid from the surrounding environment. The absorption ability can be characterized by the swelling kinetics and equilibrium swelling. The swelling kinetics generally involves assessing the time dependence of the rate with which a dry cryogel absorbs a given liquid (during isothermal regime), starting from the current liquid uptake capacity (Wu) expressed as [50,110]:

(1) $$W_{u}\;(\%)=\frac{W_{t}-{\;W}_{d}}{W_{e}}\;\times \;100\%$$

where *W_t_* is the current weight of swollen cryogel at time t, *W_d_* is the weight of dry cryogel, and *W_e_* is the weight of liquid in cryogel at its equilibrium swelling. On the other hand, the equilibrium swelling is defined by the so-called ESR as follows [50,110]:

(2) $$\mathrm{ESR}\;(g/g)=\;\frac{W_{s}-W_{d}}{W_{d}}$$

where *W_s_* is the weight of swollen cryogels at swelling equilibrium.

**Figure 7:**
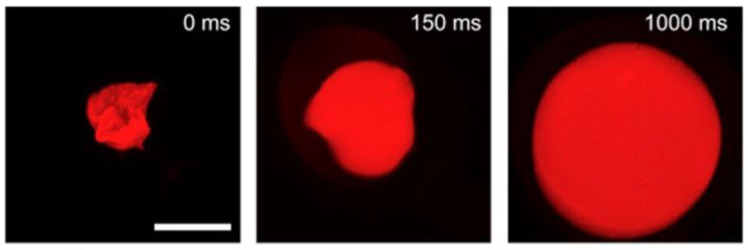
The images of collapsed gelatin cryogels (0 ms) and the images of rapid rehydration to original shape when exposed to excess phosphate buffered saline (1000 ms) [66]. Reprinted from ref. [66] with permission, © Elsevier.

Relatively recently, in order to allow a facile monitoring of the swelling process, 2-photon microscopy has been proposed. For example, Koshy et al. studied the swelling process of rhodamine-gelatin cryogels using 2-photon fluorescence imaging [66]. As can be seen in Figure 7, rhodamine-gelatin cryogels display rapid swelling behavior. This result can be attributed to the large and highly interconnected porous structure of cryogels, in which liquid can diffuse easily [111].

## 3. Biomedical Applications of CBCs and GBCs

CBCs and GBCs have excellent biological properties, such as low immunogenicity, biodegradability, biocompatibility, hydrophilicity, etc., making them suitable for biomedical applications. This section summarizes their biomedical applications in recent years, mainly including tissue engineering, cell culture, and storage.

### 3.1. Tissue Engineering

Tissue engineering is an emerging technique and its goal is to develop composite materials containing cells, scaffold, and bioactive agents to reconstruct the structural and functional properties of impaired or degenerated tissue or organ [112,113]. Generally, a desirable scaffold should have interconnected pores to support cell migration, sufficient mechanical strength to maintain the scaffold structure under contraction, and good biocompatibility and hydrophilicity to enable a proper cell adhesion and proliferation [114]. CBCs and GBCs are ideal scaffold materials for tissue engineering owing to their highly interconnected macropores, excellent biocompatibility, and adjustable mechanical properties. In recent years, a large number of publications have demonstrated the wide applications of CBCs and GBCs in the field of tissue engineering, ranging from soft-tissue to hard-tissue [115]. Table 2 illustrates some examples of the applications of CBCs and GBCs used for tissue engineering.

#### 3.1.1. Bone Tissue Engineering 

Bone is a complex tissue, playing a critical role in the body by supporting mechanical stress and maintaining ionic balance. It consists of calcium phosphate (69–80 wt%, mainly hydroxyapatite), collagen (17–20 wt%, predominantly type I collagen), and other components (water, proteins, etc.) [15,135,136]. Generally speaking, bone can regenerate and heal spontaneously when it comes to small size of defects, particularly in younger people. However, when the defects exceed a certain size limit, spontaneous bone regeneration cannot be achieved [113]. In these situations, it is necessary to use bone graft substitutes to induce the formation of new bone in defect sites [113]. Due to the limited regeneration ability of bone and the low supply of bone donors, bone tissue engineering has been proposed to treat bone defect diseases.

In bone tissue engineering, the fundamental aim is to develop a suitable scaffold, which can imitate the microenvironment of native bone in vivo, and can provide the temporary supporting matrices for the proliferation and migration of osteoblasts, or cells with osteogenic differentiation potential before the ECM produced [133,137]. Additionally, a suitable scaffold should also be biodegradable and biocompatible. Several studies have indicated that CBCs and GBCs are attractive scaffolds for bone tissue engineering owing to their interconnected macropores structure, inherent excellent biocompatibility and biodegradability, as well as the biochemical properties similar to the microenvironment of ECM [105,122]. Furthermore, the RGD sequence present in collagen and gelatin molecular structures can promote cell adhesion [138]. This property further expands the application of CBCs and GBCs in bone tissue engineering.

**Figure 8:**
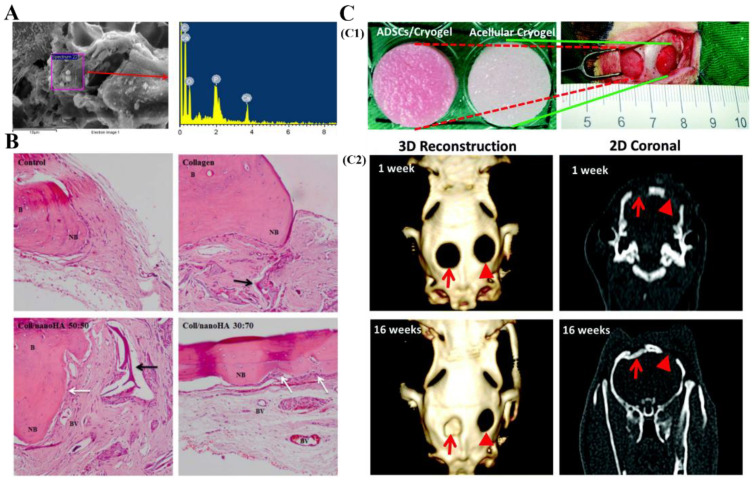
(**A**): EDS analysis of mesenchymal stem (MSCs) in gelatin/cellulose cryogels [133]; (**B**): The optical micrographs of H and E stained collagen and collagen/nonhydroxyapatite scaffold sections after 12 weeks of in vivo implantation. (B-bone tissue: NB-new formed bone: BV-blood vessel. Black arrows show the implanted material and white arrows show the osteblastic cell layer.) [104]; (**C**): The photographs of gelatin/nHAP/BMP-2 cryogels with or without rabbit ADSCs before implantation (**C1**), and the computed tomography (CT) scanning images after implanting cryogels into rabbit calvarial defects at 1 and 16 weeks (**C2**) [93]. Reprinted from refs. [93,104,133] with permission, © RSC, World Scientific and Wiley.

Zhao et al. fabricated collagen scaffolds using cryogelation technique and studied the osteogenic differentiation ability of mouse osteoblasts (MC3T3-E1) in these systems [139]. The results showed that the obtained collagen-based scaffolds with large pore size are beneficial for the proliferation and differentiation of MC3T3-E1 cells [139]. Gelatin/hyaluronic acid cryogels and gelatin/cellulose cryogels were prepared, and their effect on the osteogenesis and mineralization of adipose-derived stem cells (ADSCs) and mesenchymal stem cells (MSCs) was also investigated by Tsung and Gorgieva, proving that both ADSCs and MSCs can spread and proliferate on the surface of these cryogels [89,133]. The mineralization produced by ADSCs and MSCs experimentally observed by energy dispersive spectroscopy (EDS) (Figure 8A) confirmed the osteogenic differentiation potential of ADSCs and MSCs within GBCs. The ability of high proliferation rate and bone-specific mRNA expression of these cells adhered on cryogels proves the potential use of CBCs and GBCs in bone-tissue-engineering.

Excellent capacity of osteogenic differentiation and mineralization of cells are crucial in bone tissue engineering. Generally, pure collagen or gelatin cryogels have a low ability to induce this behavior. To further enhance the properties abovementioned, the strategies of incorporating of bioactive components, e.g., bone-morphogenetic proteins (BMPs), hydroxyapatite, bioglass, and many other useful components into CBCs and GBCs have been developed in recent years [71].

Salgado et al. found that the presence of hydroxyapatite in cryogels resulted in higher overall cellular proliferation and faster new bone formation (Figure 8B) compared to pure collagen cryogels [104,105]. Cai et al. used hydroxyapatite nanowires (HANWs) and hydroxyapatite nanorods (HANRs) as bioactive additives to fabricate GelMA/hydroxyapatite composite cryogels, and explored the effect of these cryogels on the growth of bone marrow mesenchymal stromal cells (BMSCs) [123,124]. In vitro cell culture tests showed that BMSCs growing in the HANWs and HANRs composited gelatin cryogels exhibit better osteogenic differentiation ability than BMSCs growing in the pure gelatin cryogel. In another study of Cai’s group, they found that the GelMA/HANWs cryogels can induce the Neo-bone tissues formation [140]. Hixon et al. evaluated the effect of various forms of hydroxyapatite (bone-char, and pure nanohydroxyapatite) and bioglass on the calcification potential of gelatin/chitosan cryogels [141]. Their results showed that the gelatin/chitosan cryogels loaded with bone-char exhibited increased mineralization ability.

Chen et al. have demonstrated that the addition of bone morphogenetic protein (BMP-2) into gelatin/nanohydroxyapatite (nHAP) cryogels can further improve the osteo-regeneration of rabbit ADSCs [93]. In vivo animal testing also confirmed the formation of new bone (Figure 8C) [93]. Chang et al. and Yang et al. also investigated the effect of BMPs on the osteogenic potential of gelatin/hydroxyapatite/*β*-tricalcium cryogels, and obtained similar results to Chen’s group [114,142]. In addition, growth factor has also been used to improve the osteogenesis and angiogenesis ability of collagen or gelatin-based cryogels. For example, Kim et al. incorporated the vascular endothelial growth factor (VEGF) into gelatin/heparin/whitlockite cryogels and found that the VEGF and whitlockite in these scaffolds have synergistic effects in promoting bone regeneration [91]. Similarly, VEGF was also introduced into gelatin/hydroxyapatite composite cryogels [95]. In the bone repair process, it can promote new bone formation by increasing MSCs chemotaxis and stimulating osteoblast differentiation and proliferation [95]. Furthermore, after the VEGF-loaded gelatin/hydroxyapatite cryogels were implanted in bone defects of rabbit tibiae for 6 weeks, fracture healing was clearly observed.

#### 3.1.2. Cartilage Tissue Engineering

Articular cartilage is an avascular tissue covering the bone surface in joints, and is made up of chondrocytes, progenitor cells, and ECM [143,144]. Its main function is to promote frictionless movement within articulated bones [144]. Articular chondrocytes have low density and poor cellular metabolic capacity. These inherent features of cartilage tissue contribute significantly to its limited self-repairing capability [144]. Additionally, the lager-sized un-healed cartilage defects may lead to secondary osteoarthritis, aggravating the previous defects and increasing patient sufferings [145]. Therefore, surgical intervention is required when chondral lesions are beyond critical size (>3 cm^2^) [144]. Many therapeutic approaches, including microfracture, osteochondral transplant, and autologous chondrocytes implantation, have been employed to repair large chondral damages [146]. Nevertheless, their limitations in practical application, such as site morbidity, graft hypertrophy, and inconsistent repair tissue still exist [146]. To overcome these shortcomings, cartilage tissue engineering was proposed, which provides a new remedy for damaged cartilage repair and regeneration. 

**Figure 9:**
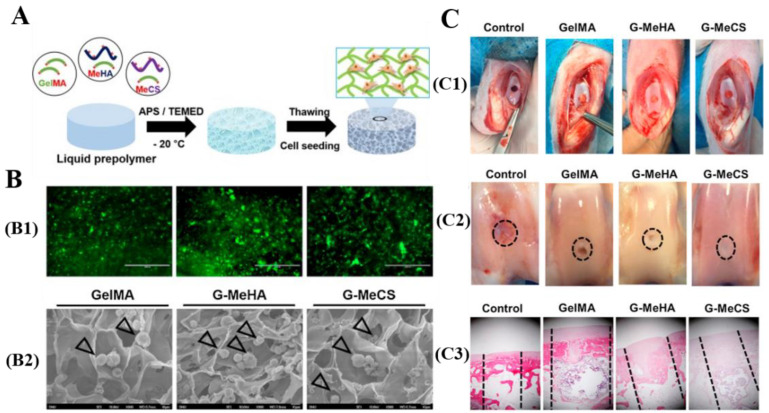
(**A**): Schematic representation of GelMA/MeHA and GelMA/MeCS cryogels fabricated by cryo-polymerization [73]; (**B**): CLSM (**B1**) and SEM (**B2**) images of cells adhered on GBCs [73]; and (**C**): Implantation of GBCs into osteochondral defect in rabbit model: (**C1**) the images of rabbit defects filled with acellular GBCs; (**C2**) the images of defect surfaces after 8 weeks of implantation of GBCs; and (**C3**) the images of hematoxylin and eosin staining analysis of rabbit knees after implantation of GBCs into osteochondral defect in a rabbit model [73]. Reprinted from ref. [73] with permission, © Elsevier.

To develop scaffolds for cartilage tissue engineering, the porous structures are crucial to support chondrocytes migration and proliferation within the construct. CBCs and GBCs can be used as scaffolds for cartilage tissue engineering due to their interconnected macroporous network structures and biochemical features, similar to native cartilage ECM. Specifically, Chen et al. and Lin et al. have demonstrated that the gelatin/chondoitin-6-sulfate/hyaluronan (GCH) cryogels can mimic the microenvironment of natural cartilage ECM to induce cell adhesion and proliferation [126,147]. Moreover, their studies showed that incorporation of chitosan into GCH-cryogels significantly increases the secretion of glycosaminoglycans (GAGs) and type II collagen. Type II collagen is the important component of native cartilage ECM and represents the marker for chondrocytes differentiated [126,148]. Han et al. fabricated gelatin cryogels by copolymerization of GelMA with methacrylated hyaluronic acid (MeHA) or methacrylated chondroitin sulfate (MeCS) (Figure 9A) [73]. Confocal laser scanning microscopy (CLSM) and SEM analysis of in vitro cells culture showed that chondrocytes adhered, infiltrated, and proliferated within the interconnected macroporous structure of the composite scaffolds, and maintained their spherical morphology (Figure 9B). Han et al. also found that the introduction of MeHA and MeCS into GelMA significantly enhances the accumulation of proteoglycans and gene expression of type II collagen. Additionally, in comparison with GelMA and GelMA/MeHA cryogels, GelMA/MeCS cryogels can markedly induce the neo-cartilage formation, and can be fully integrated with the surrounding host tissue after implantation into the rabbit osteochondral defects (Figure 9C).

The addition of cell-derived bioactive molecules in CBCs and GBCs also can improve the proliferation of chondrocytes. Kumar’s group synthesized the chitosan–agarose–gelatin (CAG) cryogels loaded with transforming growth factor-*β*1 (TGF-β1) [149]. Subsequently, the composite CAG-cryogels were implanted in the subchondral cartilage defect of New Zealand rabbits to evaluate their potential for cartilage repair. Their results demonstrated that combining TGF-β1 and CAG-cryogels with chondrocyte can enhance the healing ability and rate of cartilage, compared to single use of TGF-β1. These studies prove the attractiveness of CBCs and GBCs as scaffolds for cartilage tissue engineering.

#### 3.1.3. Skin Tissue Engineering/Wound Healing

Skin, one of the largest tissues in vertebrates, is composed of tough epidermis (mainly keratinocytes) and relatively acellular dermis (a collagen-rich ECM) [150]. Skin is regarded as a physical protective barrier at the interface between body and external environment to shield the body from the insults of pathogens and microorganisms [151]. Large area loss and irreversible damage of skin caused by burns, chronic wounds, or traumatic accidents can seriously affect the living quality of patients. Accordingly, surgical intervention is required to assist wound healing and skin regeneration. The traditional methods used to treat skin wounds primarily include wound dressing, xenografts, autografts, and allographs [151]. However, the limitations of these methods, such as antigenicity and insufficient skin regeneration ability, reduce their clinical applications for skin repair [151]. To solve this challenge, skin tissue engineering is a promising and useful approach.

**Figure 10:**
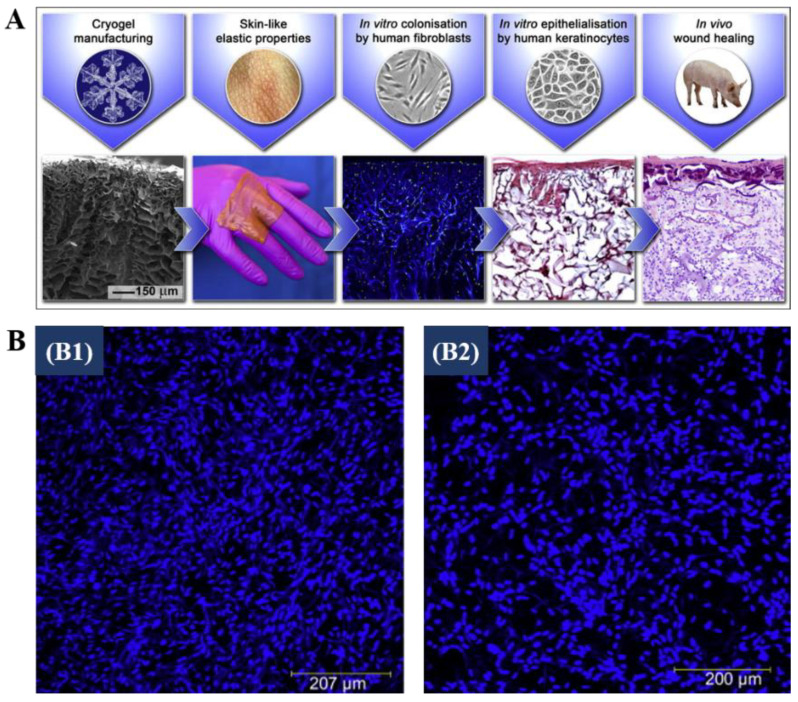
(**A**): Schematic representation of gelatin cryogels with attached silicone layer used for skin tissue engineering [152]; (**B**): CLSM images of primary human dermal fibroblasts cultured on a gelatin/fibrinogen cryogel (**B1**), and Integra^®^ (**B2**), for 5 days [49]. Reprinted from refs. [49,152] with permission, © Elsevier.

With their interconnected structure of macropores enhancing cells migration, and their high liquid absorptive capacity preventing liquid accumulation in the wound, cryogels have been considered as promising substitutes for skin tissue engineering and wound healing [11,138]. In particular, several studies have focused on using CBCs and GBCs as skin substitution materials, as they can provide the ECM microenvironment for the attachment, proliferation, and differentiation of dermal fibroblasts cells. For example, Shevchenko et al. fabricated gelatin cryogels onto the surface of the thin silicon-based nonporous film to obtain the double-layer cryogel-containing sheets gelatin cryogels, and evaluated their application in vitro cell culture and in vivo wound healing (Figure 10A), suggesting a favorable role played by these materials as substitution for skin [152]. Allan et al. developed a novel matrix composed of gelatin and fibrinogen for wound healing [49]. The resulting cryogels possessed high porosity (about 90%) and interconnected porous structure with pore size up to 120 μm [49]. Compared to the primary dermal fibroblasts (SKF371) seeded on commercial Integra^®^ artificial skin, the cells seeded on gelatin/fibrinogen cryogels showed higher cell density after 5 days of culturing (Figure 10B) [49]. During the wound healing process in vivo, the extent of cellular infiltration in scaffolds played a crucial role. Allan et al. also assessed the infiltration rate of fibroblasts into gelatin/fibrinogen cryogels [88]. They found that more fibroblasts were present on the exterior of gelatin/fibrinogen cryogels than on the interior. Moreover, they discovered that the infiltration extent of fibroblasts was positively related to the fibrinogen content in the cryogels. These findings provide references for the application of CBCs and GBCs in skin tissue engineering.

Tyeb et al. designed a novel composite cryogels made of gelatin, sericin and laminin with applicability for the culture of fibroblasts, keratinocytes, ADSCs, and HUVEC cells [92]. Additionally, when the composite cryogels loaded with ADSCs were implanted in diabetic rats, these scaffolds showed enhanced wound healing capacity and mild inflammation [92]. Recently, GBCs have been investigated to imitate the microenvironment of different skin layers. For instance, Priya et al. explored the potential of a bilayer cryogel, with gelatin as regenerative layer, in the treatment of acute skin wound [116]. Fluorescence microscopy and SEM images showed that the gelatin cryogels layer can support the infiltration, attachment, and proliferation of fibroblasts and keratinocytes [116]. In adition, the results indicated that this bilayer cryogel has enhanced wound healing capacity after implanting it in rabbits’ skin defects, compared to untreated rabbits.

#### 3.1.4. Vascular Tissue Engineering

Vascularization is of crucial importance for the bone, heart, liver, and skin tissue repair, as it can provide nutrients and oxygen. Therefore, vascular network regeneration has become one of the primary targets for the development of tissue engineering in recent years. It has been reported that GBCs can serve as the cell matrices for vascular tissue regeneration. For example, an early study conducted by Vrana et al. demonstrated the applicability of PVA/gelatin cryogels for vascular tissue engineering [120,121]. They reported that bovine arterial endothelial cells adhered and proliferated on PVA/gelatin cryogels. In addition, they found that applying shear stress can promote neo-endothelialization on this scaffold. Similarly, GBCs were loaded with bioactive compounds to enhance the capacity of vascularization. Kimet. et al. studied the properties of heparin/gelatin cryogels loaded with VEGF and assessed the vascularization potential of this cryogel in animals, pointing out that VEGF favorably contributed to the host cells migration and, eventually, enhancing vascularization [90].

#### 3.1.5. Neural Tissue Engineering and Other Tissue Engineering

Nerve injuries may give rise to the patient with a permanent disability of cognitive, motor, or psychotic functions, profoundly affecting patient’s quality of life [153,154]. Autologous nerve grafts are often not readily available, as the supply of nerves is limited [153]. Allogenic grafts would lead to the inflammatory immune response [153,154]. The emerging tissue engineering in recent years provides a new alternative for nerve injuries treatment. CBCs and GBCs have been studied as scaffolds for neural tissue engineering given their good biocompatibility, biodegradability, high porosity, and interconnected macropores.

**Figure 11:**
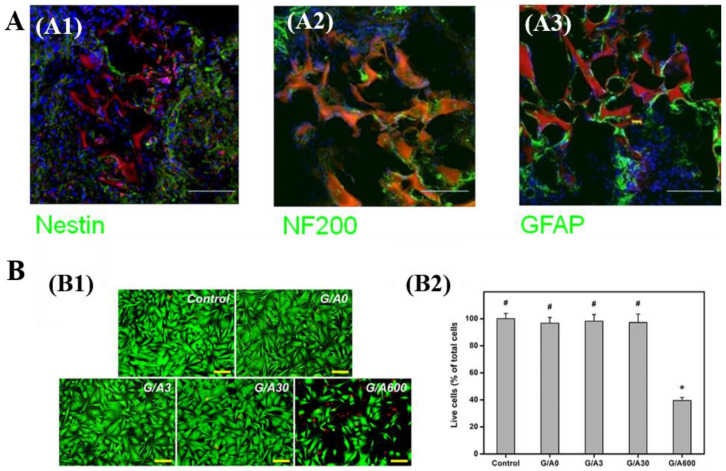
(**A**): CLSM images of gelatin/laminin cryogels integrated with host brain tissue ((**A1**): Nestin; (**A2**): NF200; and (**A3**): GFAP) after implantation into the rat brain [128]; (**B**): (**B1**) Fluorescence microscopy images of rabbit corneal keratocyte cultured in control group and various gelatin/ascorbic acid cryogels (i.e., G/A0, G/A3, G/A30 and G/A600) for 2-day; (**B2**) Mean percentage of living cells after seeding rabbit corneal keratocyte on various gelatin/ascorbic acid cryogels and then incubating for 2 days at 37 °C; * *p* < 0.05 vs. all groups; ^#^ *p* < 0.05 vs. G/A600 groups [101]. Reprinted from refs. [101,128] with permission, © Elsevier.

In a study by Agarwal et al., bone marrow mesenchymal stem cells (BM-MSCs) were cultured on collagen cryogels crosslinked by amino-functionalized graphene to study the neural tissue regeneration capacity [24]. Thus, BM-MSCs growth on collagen cryogels exhibited an enhanced mRNA expression of neuronal markers, MAP-2 kinase, β tubulin III and nestin, which demonstrates the capacity of these crosslinked systems to support the neuronal differentiation of BM-MSCs [24]. Jurga et al. found that gelatin/laminin cryogels could be used to culture human cord blood-derived stem cells (hCBSCs), and contribute to the hCBSCs neuronal differentiation and the formation of neural niche-like structures [128]. The transplantation experiments on rats showed that the host neuroblasts can migrate and penetrate gelatin/laminin cryogels (Figure 11A) [128]. Additionally, the cryogels can integrate with host NF200-positive neuroblasts (Figure 11A) [128]. Singh et al. fabricated gelatin/chitosan cryogels and applied them in peripheral neural regeneration [130]. In vitro results proved that the cryogels can facilitate the proliferation of Neuro 2a cells and BM-MSCs and the regeneration of nerve. In another study conducted by Vishnoi et al., gelatin/chitosan/polypyrrole cryogels were prepared to mimic the in vivo microenvironment [131]. At the same time, implantation of this multicomponent cryogel in a critical size sciatic nerve defect (1.5 cm), intentionally created in rats, was investigated regarding its regenerative effect [131].

CBCs and GBCs have also displayed the potential for application in adipose, cardiac, and corneal stromal tissue engineering. For example, Chen et al. utilized gelatin/hyaluronic acid cryogels as cell scaffolds and applied it in adipose tissue engineering [99]. In vitro and in vivo experimental results showed that gelatin/hyaluronic acid cryogel provided a favorable microenvironment for cell attachment and proliferation and enhanced the ADSCs adipogenesis [99]. Luo et al. fabricated gelatin/ascorbic acid cryogels and evaluated the effect of ascorbic acid on the application potential of gelatin/ascorbic acid cryogels in corneal stromal tissue engineering [101]. Experimental results suggested that gelatin cryogels with optimum ascorbic acid dosage can promote keratocytes proliferation and matrix regeneration. However, gelatin cryogels with higher ascorbic acid dosage can lead to cytotoxicity, with a negative impact on corneal keratocytes proliferation (Figure 11B) [101]. Similar findings were reported by Sazwi et al. and Chularojmontri et al. [155,156]. Wang et al. have demonstrated the potential of a methacrylated gelatin (GelMA)-poly (ethylene glycol) diacrylate (PEGDA) cryogels as an artificial patch for cardiac tissue engineering [134].

### 3.2. Other Biomedical Applications

#### 3.2.1. Cell Culture

The extracellular matrix (ECM) is a vital significant constitution of the cellular microenvironment, playing a critical role in regulating cellular behavior and function, such as adhesion, proliferation, and differentiation [157]. Cryogels prepared from collagen and gelatin have similar characteristics to natural ECM. This is why cryogels of the abovementioned compositions have been developed as scaffolds for cell culture [158,159].

**Figure 12:**
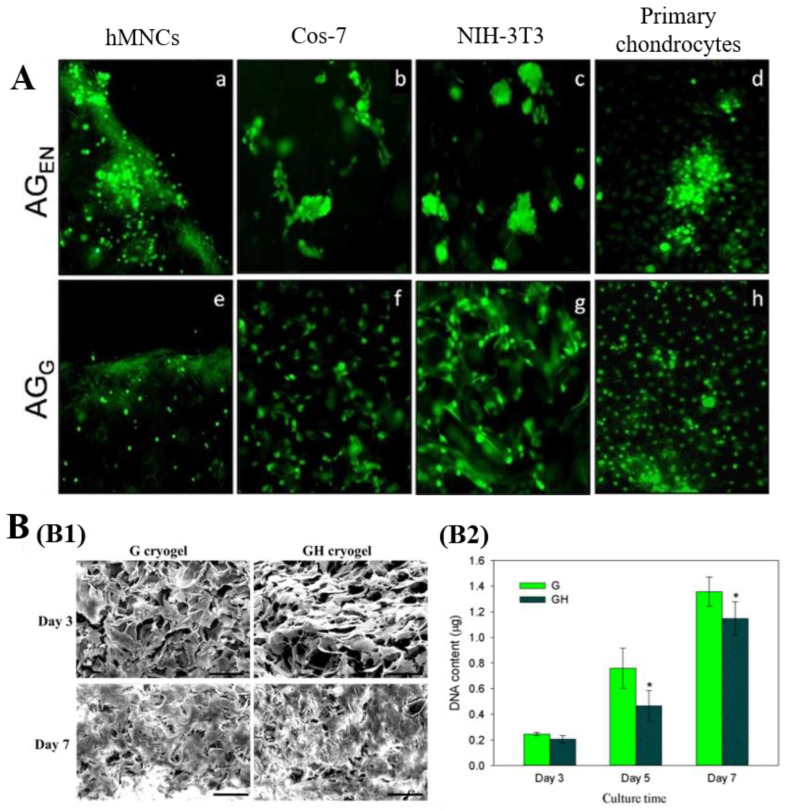
(**A**): The viability and growth patterns of four different cell types (i.e., hMNCs (**a**,**e**), Cos-7 (**b**,**f**), NIH-3T3 (**c**,**g**) and primary chondrocytes (**d**,**h**) in alginate-gelatin-glutaraldehyde cryogels (AG_G_) and alginate-gelatin-EDC-NHS cryogels (AG_EN_) [160]; (**B**): (**B1**) SEM images of mesothelial cell morphology within gelatin (G) or gelatin/ hyaluronic acid (GH) cryogels. (**B2**) the cell proliferation rate was determined by DNA assays; * *p* < 0.05 compared with G [161]. Reprinted from refs. [160,161] with permission, © Wiley and MDPI.

A previous study reported the applications of collagen cryogels for cell culture [102]. The experimental findings indicated that adipose mesenchymal stem cells (AD-MSCs) seeded on collagen cryogels displayed an increasing ability of viability and proliferation compared to AD-MSCs cultured in traditional cell culture plates [102]. Kumar’s group conducted a series of studies to explore the ability of GBCs to be used as substrates for cell culture. For instance, chitosan/agarose/gelatin and carrageenan/gelatin cryogels were prepared, and the functional activities of fibroblasts Cos-7 seeded on these gels were studied [86,97]. The Cos-7 cells displayed a good adherence and proliferation together with the production of new ECM on these cryogel matrices [86,97]. Umbilical cord blood derived mononucleated cells (hMNCs), fibroblasts (Cos-7 and NIH-3T3), and primary chondrocytes were seeded into alginate/gelatin cryogels to examine the cell adhesion and growth behavior in the matrices [160]. All types of cells were homogenously attached and uniform growth on the surface of alginate/gelatin cryogels crosslinked by glutaraldehyde after 5 days of culture (Figure 12A) [160]. However, they exhibited uneven adhesion and aggregated growth on the surface of alginate/gelatin cryogels crosslinked by EDC/NHS (Figure 12A) [160]. Additionally, Kao et al. fabricated gelatin/hyaluronic acid cryogels to culture mesothelial cells and evaluated the impact of hyaluronic acid on cellular morphology and proliferation [161]. Results showed that introduction of hyaluronic acid caused changes in cell morphology and a reduction in cell proliferation rate (Figure 12B) [161].

#### 3.2.2. Cell Transportation and Cryopreservation

**Figure 13:**
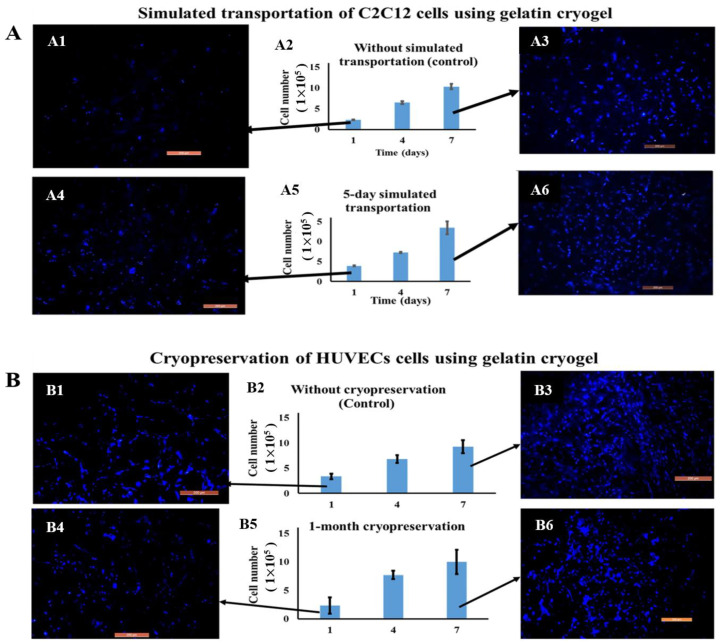
(**A**): The cell viability and fluorescence microscopy analysis of C2C12 cell seeded gelatin cryogels without simulated transportation (**A1**–**A3**) and after 5 days of simulated transportation (**A4**–**A6**) [163]; (**B**): The cell viability and fluorescence microscopy images of HUVECs cells seeded on gelatin cryogels without simulated cryopreservation (**B1**–**B3**) and after 1 month of simulated cryopreservation (**B4**–**B6**) [163]. Reprinted from ref. [163] with permission, © Wiley.

Apart from cell culture, there have been several studies highlighting the critical roles of GBCs in cell storage. In a previous study, PVA/gelatin cryogels were prepared as matrices for cell cryo-storage [162]. The concluding remark was that the viability of vascular smooth muscle cells was sustained after 2 weeks of cryopreservation in liquid nitrogen. In another study, the potential of gelatin cryogels as transporting matrices for mouse myoblast cells (C2C12) and cryo-storage matrices for C2C12, human hepatocellular liver carcinoma cell line (HepG2), and human umbilical vein endothelial cells (HUVECs) was investigated by Kumari and Kumar [163]. They reported that C2C12 cells can successfully retain their viability after 5 days of simulated transportation (Figure 13A), and these cells seeded on gelatin cryogels were able to proliferate after 1 month of simulated cryopreservation (Figure 13B) [163]. This research proves the feasibility of GBCs in the fields of cell transportation and cryopreservation.

## 4. Conclusions and Future Perspectives

Collagen and gelatin are rated among the top macromolecule candidates for the fabrication of cryogels. Generally, the properties of CBCs and GBCs, such as pore shape and size, mechanical characteristics, swelling behavior, biocompatibility, and biodegradability, are important in practical applications. These peculiarities are closely related to the preparation methods, freezing rate and temperature, and collagen or gelatin concentration. It has been widely reported that cross-linking is always needed to improve their mechanical properties and thermal stability, while also controlling their degradation rate. Although chemical cross-linking induced by small molecules is proven to be very effective in enhancing the toughness or robustness of these cryogels, the cytotoxicity of residual chemical reagents is inevitable. From the viewpoint of safety and cost-effectiveness, the macromolecule cross-linking could be an alternative approach. Additionally, the newly proposed free-radical polymerization is emerging as a very promising strategy for the stabilization of CBCs and GBCs. Furthermore, the mechanical properties of these cryogels can be further improved by increasing the repeated freezing–thawing cycles. Other additional and in-depth studies are still needed to have a comprehensive understanding of these cryogels preparation, structures, and functions.

As for practical applications, CBCs and GBCs have become very attractive materials in biomedicine fields, such as tissue engineering, wound dressing, cell culture, and cell storage, as they possess unique sponge-like morphology, high porosity and interconnected large pores, and excellent biocompatibility and biodegradability. Incorporating bioactive components into CBCs and GBCs is always necessary to achieve specific biological functions. The technological advances in well-designed in vitro and in vivo assays will allow deeper insight and better understanding of the physicochemical properties, bioactivity, and biological involvement of these cryogels. As such, their applications in biomedical fields will be further expanded on the basis of related fundamental research.

## Figures and Tables

**Table 1:** An overview on cryogels based on gelatin/collagen.

Polymers Added to Collagen/Gelatin	Crosslinker/Initiator	Temperature of Cryotropic Gelation (°C)	References
Gelatin/PVA	None/Glutaraldehyde	−12 °C/−16 °C	[51,76,77]
Gelatin/PAN	Glutaraldehyde	−12 °C	[78]
GelMA/PPY	None	−24 °C	[79]
Gelatin/PNIPAAm	Glutaraldehyde	−12 °C	[80]
Gelatin/Agarose	Glutaraldehyde	−12 °C	[81]
Gelatin/Chitosan	Glutaraldehyde/Oxidized dextran	−20 °C/−12 °C	[50,82,83,84,85]
Gelatin/Carrageenan	EDC/Glutaraldehyde	−12 °C	[86,87]
Gelatin/Fibrinogen	Glutaraldehyde	−12 °C	[49,88]
Gelatin/Nanocellulose	Dialdehyde starch	4 °C	[27,89]
Gelatin/Heparin	EDC/NHS	−20 °C	[90,91]
Gelatin/Sericin	Glutaraldehyde	−12 °C	[92]
Gelatin/Hydroxyapatite	EDC/Glutaraldehyde/Oxidized dextran	−20 °C	[64,93,94,95]
Gelatin/Chitosan/Agarose	Glutaraldehyde	−12 °C	[96,97,98]
Gelatin/Hyaluronic acid	EDC	−20 °C	[99]
Gelatin/Alginate	No	−15 °C	[100]
Gelatin/Ascorbic acid	EDC	−20 °C	[101]
Collagen/polydopamine	EDC	−20 °C	[102]
Collagen/calcium peroxide	EDC	−20 °C	[103]
Collagen/graphene	EDC/NHS	−12 °C	[24]
Collagen/hydroxyapatite	EDC	−18 °C	[104,105]

**Table 2:** Reported examples of CBCs and GBCs used for tissue engineering.

Polymers	Cell Type	Tissue	Reference
Gelatin + Fibrinogen	Human dermal fibroblasts	Skin	[49,88]
Gelatin + Sericin + Laminin	Adipose-derived stem cell		[92]
Gelatin + Polyvinylpyrrolidone-iodine	Fibroblasts, Keratinocytes		[116]
Gelatin + Collagen + Hyaluronic acid	Human skin cells		[117]
Gelatin + Poly (vinyl alcohol)	Fibroblasts		[77]
Gelatin + Polymethyl methacrylate	Adipose-derived stem cell		[118]
Gelatin + Alginate/Hyaluronic acid	None		[119]
Gelatin + Pectin + Transition metal	Fibroblasts		[42]
Gelatin + Poly (vinyl alcohol)	Endothelial cells	Vascular	[120,121]
Gelatin + Heparin	Human umbilical vein endothelial cells	Bone	[90]
Gelatin + Chitosan	Bone osteosarcoma-derived cells		[122]
GelMA + Hydroxyapatites	Bone marrow mesenchymal stromal cells		[123,124]
Gelatin + Hydroxyapatite + Vascular endothelial growth factor	None		[95]
GelMA + Bioglass	Human tonsil-derived mesenchymal stem cells		[71]
Collagen/nanohydroxyapatite	Human bone marrow stromal cells		[104,105,125]
Gelatin + Chondroitin-6-suifate + hyalueonan	Chondrocytes	Cartilage	[126]
GelMA + Mecs	Chondrocytes		[73]
Gelatin + Hyaluronic acid	Chondrocytes		[127]
Gelatin + Laminin	Human cord blood-derived stem cells	Neural	[128]
GelMA + methacrylated hyaluronic acid	rabbit Schwann cells		[129]
Gelatin + Chitosan	Neuro 2a cells bone, Bone marrow stem cells		[130]
Gelatin + Chitosan + Polypyrrole	Bone marrow stem cells		[131]
Gelatin	NIH-3T3 cells		[132]
Gelatin + Haluronic acid	Adipose-derived stem cells	Adipose	[99,133]
Gelatin + Ascorbic acid	Corneal keratocytes	Corneal	[101]
GelMA + poly(ethylene glycol) diacrylate	None	Cardiac	[134]

## Data Availability

Not applicable.
